# Prevalence and characterization of β-lactamase-producing bacteria in gingivitis among diabetic and non-diabetic patients: a comparative microbiological study

**DOI:** 10.1099/acmi.0.001094.v3

**Published:** 2025-10-03

**Authors:** Mohanned Mohamed Alwashsiah, Asma Abdellatif Abbas

**Affiliations:** 1 Microbiology Department, Faculty of Pharmacy, Misurata University, Misurata, Libya

**Keywords:** antibiotic resistance, *β*-lactamase, diabetes mellitus, gingivitis, oral microbiota

## Abstract

**Background.** Gingivitis is a reversible gingival inflammation that may progress to periodontitis if untreated. Diabetes mellitus alters the oral microbiota and weakens host defenses, increasing susceptibility to infection.

**Objectives.** To investigate the prevalence and characterization of *β*-lactamase-producing bacteria isolated from gingival swabs of diabetic and non-diabetic patients with clinically confirmed gingivitis.

**Methods.** Thirty-seven patients were enrolled (17 diabetics and 20 non-diabetics). Gingival swabs were cultured and identified by conventional microbiological and analytical profile index (API). Antimicrobial susceptibility was tested according to the Clinical and Laboratory Standards Institute 2023 guidelines. *β*-Lactamase activity was assessed using an iodometric colourimetric assay.

**Results.** A total of 65 bacterial isolates were obtained from 37 gingivitis patients. Polymicrobial infections predominated in diabetics (82.4%) vs. non-diabetics (45.0%). Non-diabetics were mainly colonized by *Streptococcus mutans* (45.9%) and *Staphylococcus aureus* (40.5%), while diabetics harboured more Gram-negative species, particularly *Pseudomonas aeruginosa* (21.4%) and *Enterobacteriaceae* (46.4 % vs. 2.7 %). Overall, 67.7 % of isolates were *β*-lactamase producers. Resistance was highest to ampicillin (92.3%) and amoxicillin–clavulanate (84.6%), whereas ciprofloxacin (89.2%) and piperacillin–tazobactam (78.5%) retained the greatest activity.

**Conclusions.** Diabetes is associated with increased microbial diversity, Gram-negative colonization, and a frequency of *β*-lactamase-producing bacteria in gingivitis. These findings highlight diabetes as a risk factor for resistant oral infections and underscore the need for antimicrobial stewardship, resistance surveillance and future molecular studies to clarify resistance mechanisms in high-risk groups.

## Data Summary

The data analysed in this study, including demographic variables (gender, age and diabetic status), were obtained from patient records and microbiological samples collected at the Libyan Dental Centre and the Diabetic and Endocrinology Centre in Ras Al-Tuba. All analyses were conducted on-site in compliance with institutional data protection policies. Raw data were not transferred outside the facility, and all biological samples were securely disposed of 1 year after collection in accordance with the institution’s ethical regulations.

Due to these restrictions, the supporting raw data are not publicly available. However, all results presented in this manuscript are based on anonymized and aggregated data derived from the internal analysis. No molecular or sequencing methods were performed in this study.

## Introduction

Gingivitis is a mild and reversible inflammatory disease of the gingival tissue primarily caused by the accumulation of dental plaque (biofilm) on tooth surfaces [1]. If left untreated, gingivitis can progress to periodontitis, resulting in irreversible destruction of periodontal tissues and eventual tooth loss [2]. The condition is highly prevalent across age groups and populations, particularly among individuals with poor oral hygiene, unhealthy diet or systemic diseases [3].

The microbial biofilm plays a pivotal role in the initiation and progression of gingivitis. Common bacterial genera implicated include *Streptococcus*, *Fusobacterium*, *Actinomyces* and *Treponema* [1]. Systemic conditions such as diabetes mellitus further exacerbate gingival and periodontal inflammation. Poorly controlled diabetes impairs immune responses and increases susceptibility to oral infections, thereby altering the composition of the oral microbiota [4].

Epidemiological studies have consistently demonstrated a strong association between diabetes and periodontal diseases. Diabetic patients are more likely to harbour a diverse and pathogenic oral microbiota, a situation complicated by the emergence of antimicrobial resistance [5]. Of particular concern is the production of *β*-lactamase enzymes by oral bacteria, which inactivate *β*-lactam antibiotics and reduce the effectiveness of standard treatments [6,7].

The present study was undertaken to compare the microbial profiles and the prevalence of *β*-lactamase-producing bacteria among diabetic and non-diabetic patients with clinically confirmed gingivitis. By characterizing bacterial isolates and their antimicrobial resistance patterns, this research aims to provide insights into the interplay between diabetes, oral microbial ecology and antibiotic resistance.

## Methods

This comparative cross-sectional study was conducted between November and December 2022 in Libya. A total of 37 patients with clinically confirmed gingivitis were enrolled, comprising 20 non-diabetic individuals (aged 9–65 years) recruited from the Libyan Dental Center and 17 diabetic patients (aged 12–75 years) recruited from the Diabetic and Endocrinology Center in Ras Al-Tuba.

### Sample size justification

This study enrolled 37 patients during a short window (November–December 2022). The sample size reflects feasibility constraints, including strict eligibility criteria, on-site laboratory processing and limited laboratory resources. Given these limitations, the study was designed as exploratory, and results should be interpreted with caution. Larger multicentre studies have reported substantially bigger cohorts (≥100 patients) [8], and therefore, our findings are best regarded as preliminary benchmarks to inform future adequately powered research.

### Inclusion and exclusion criteria

Patients of both sexes and all age groups presenting with clinical signs of gingivitis. Inclusion criteria: patients diagnosed with gingivitis by a periodontist based on standard clinical criteria (bleeding on probing, gingival index >1 and the absence of clinical attachment loss). Exclusion criteria: patients who had received antibiotics or antiseptic mouth rinses within the last 3 months, smokers, pregnant or lactating women and patients with systemic diseases other than diabetes mellitus.

### Sample collection and processing

Oral swabs were collected from the gingival margin of the lower right or left quadrants using sterile cotton swabs by trained dental practitioners. Samples were transported in normal saline and processed within 2 h.

### Bacterial cultivation and identification

Samples were cultured on blood agar, chocolate agar, MacConkey agar and Mueller–Hinton agar and incubated aerobically at 37 °C for 24 h. Initial identification was based on Gram staining, colony morphology and biochemical tests (citrate, indole, triple sugar iron, catalase and coagulase). Final confirmation of bacterial isolates was achieved using the API 20E and API 20 Strep identification systems [9]. This step complemented the initial biochemical and morphological tests, providing species-level accuracy and reproducibility.

### Antimicrobial susceptibility testing

Antibiotic susceptibility was determined using the disc diffusion method on Mueller–Hinton agar. Inoculum was standardized to a 0.5 McFarland turbidity standard. Antibiotics tested included ciprofloxacin, kanamycin, piperacillin–tazobactam, ampicillin and amoxicillin–clavulanic acid. Results were interpreted according to the Clinical and Laboratory Standards Institute guidelines, 2023 [10].

### Detection of *β*-lactamase production

*β*-Lactamase activity was detected using an iodine-based colourimetric assay. This method, originally adapted for the routine detection of *β*-lactamase across different *β*-lactam substrates, has been extensively validated for its applicability and reliability in microbiological studies [11]. Comparative evaluations have demonstrated that the iodometric assay provides sensitivity and specificity comparable to chromogenic methods, while offering practical advantages in low-resource laboratory settings [12]. Furthermore, its successful application in oral microbiology research supports its relevance for the present study [13].

### Statistical analysis

Statistical analyses were performed using SPSS version 27 (IBM Corp., Armonk, NY, USA) and R version 4.2.2 (R Core Team, 2022).

Descriptive statistics were presented as frequencies, percentages, means and standard deviations. Categorical variables (e.g. prevalence of *β*-lactamase production between diabetic and non-diabetic groups) were compared using chi-square, and when expected cell counts were small, Fisher’s exact test was applied, as appropriate. Continuous variables were analysed using Student’s t-test or ANOVA. Pearson’s correlation test was applied to evaluate the association between microbial load and resistance rates. A *P*-value<0.05 was considered statistically significant [14].

Given the exploratory nature and limited sample size, no *a priori* power calculation was performed; results are presented with exact tests and 95% confidence intervals where appropriate.

## Results

A total of 37 patients with clinically confirmed gingivitis were included, comprising 17 diabetic and 20 non-diabetic individuals. Bacterial growth was obtained from all swab samples.

### Patient age distribution by diabetic status

The age distribution differed significantly between groups (Table 1). Most non-diabetic cases were <20 years (12/20; 60.0%), whereas most diabetic cases were 41–69 years (10/17; 58.8%). Patients ≥70 years were represented only among diabetics (3/17; 17.6%), as shown in Table 1.

**Table 1.** Distribution of patients with gingivitis by age group and diabetic status

Age group (years)	Diabetic (*n*=17)	%	Non-diabetic (*n*=20)	%
<20	1	5.9	12	60.0
21–40	3	17.6	7	35.0
41–69	10	58.8	1	5.0
≥70	3	17.6	0	0.0

n, number; %, percentage.

Pearson’s *χ*² = 21.17, df=3, *P*<0.001. Age distribution differs significantly by diabetic status, with younger ages predominating in non-diabetics and middle-older ages in diabetics.

### Monomicrobial vs. polymicrobial infections

**Fig. 1.**
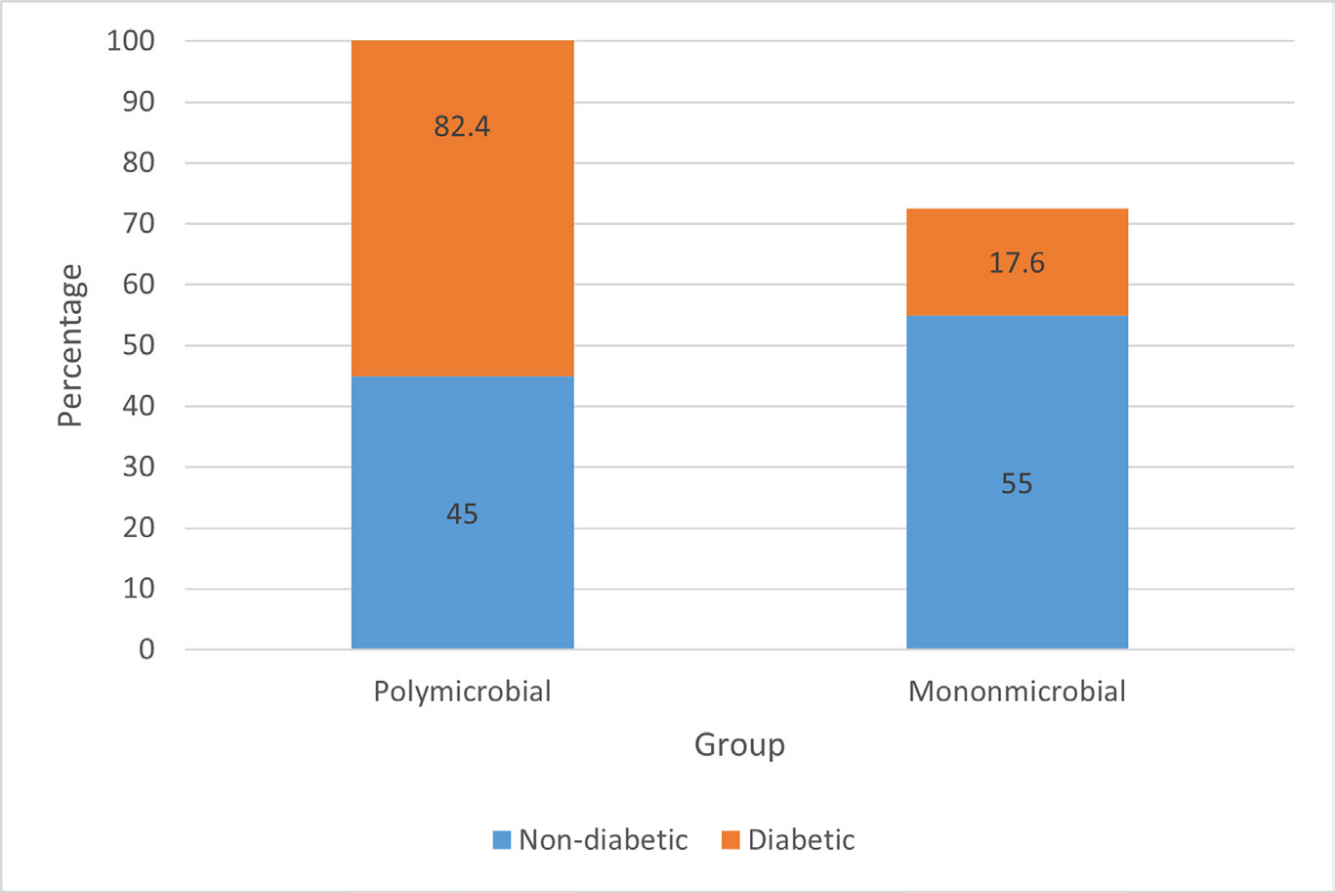
Distribution of monomicrobial and polymicrobial infections among diabetic and non-diabetic gingivitis patients. Polymicrobial infections were significantly more frequent in diabetic patients than in non-diabetic patients, highlighting the association between diabetes and complex microbial colonization in gingivitis.

Analysis of microbial diversity revealed a clear difference in the distribution of monomicrobial and polymicrobial infections between the two study groups. Among non-diabetic patients with gingivitis (*n*=20), 11 cases (55.0%) presented with monomicrobial infections, while 9 cases (45.0%) exhibited polymicrobial colonization. In contrast, the diabetic group (*n*=17) showed a predominance of polymicrobial infections, with 14 patients (82.4%) harbouring more than one bacterial isolate, compared to only 3 patients (17.6%) with monomicrobial infections, as shown in Fig. 1.

### Bacterial species distribution

A total of 65 bacterial isolates were recovered from 37 gingivitis patients. Among non-diabetic patients, the predominant bacteria were *Streptococcus mutans* (17/37 isolates; 45.9%) and *Staphylococcus aureus* (15/37; 40.5%), with *Bacillus* spp. contributing only a minor fraction (4/37; 10.8%). Virtually no Gram-negative bacilli were detected in this group, with the sole exception of a single *Pseudomonas aeruginosa* isolate (2.7%).

**Fig. 2.**
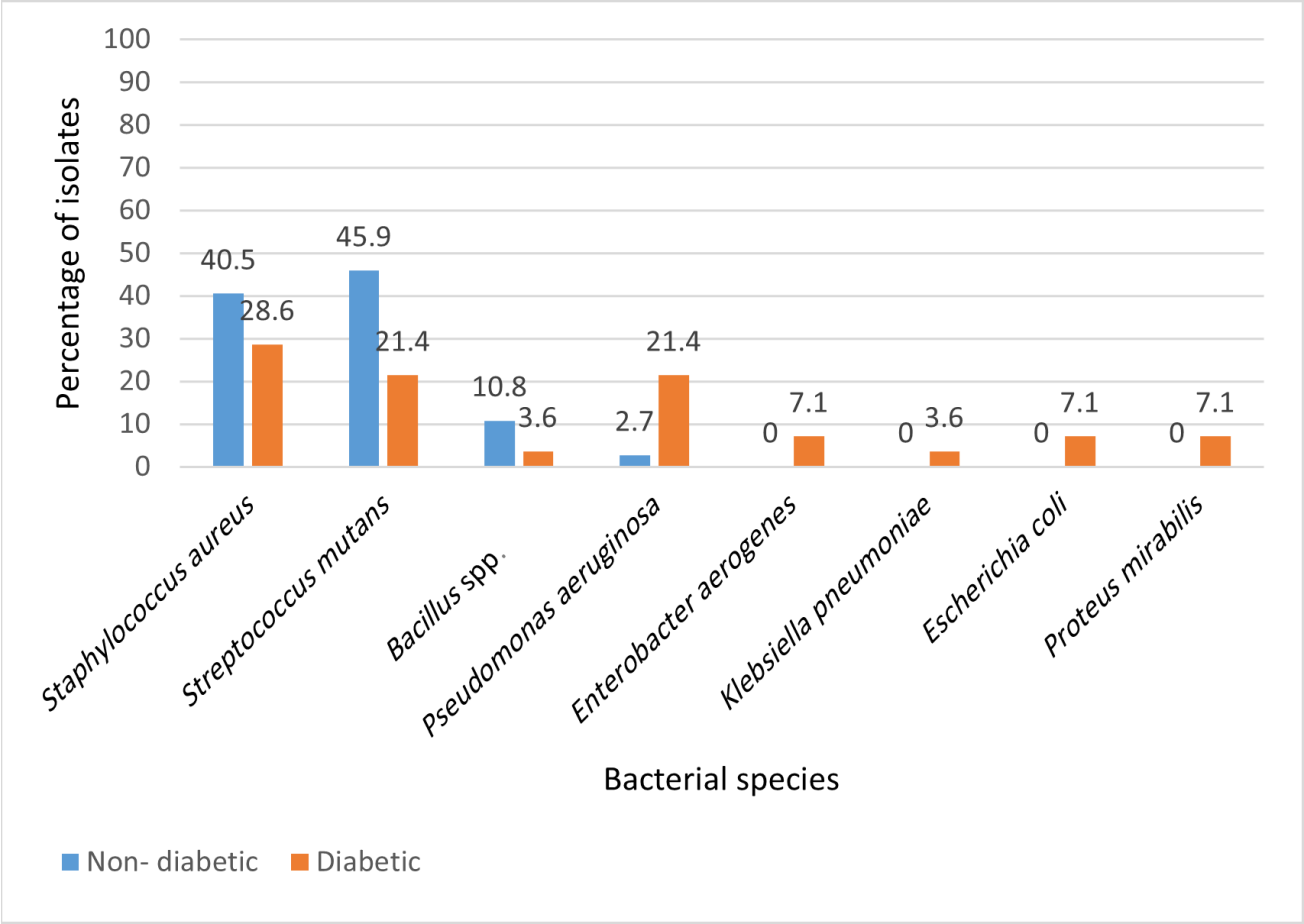
The distribution of bacterial isolates revealed distinct microbial patterns between diabetic and non-diabetic patients. Diabetic patients showed a higher prevalence of Gram-negative bacilli, particularly *Enterobacteriaceae*, compared with non-diabetic counterparts (*P*<0.05, chi-square test).

In contrast, diabetic patients showed a more diverse microbial profile. Although *Staphylococcus aureus* (8/28; 28.6%) and *Streptococcus mutans* (6/28; 21.4%) remained common, a striking feature was the emergence of Gram-negative species. *Pseudomonas aeruginosa* accounted for 21.4 %(6/28), followed by *Escherichia coli* (2/28; 7.1%), *Proteus mirabilis* (2/28; 7.1%), *Enterobacter aerogenes* (2/28; 7.1%) and *Klebsiella pneumoniae* (1/28; 3.6%). Thus, Gram-negative isolates collectively represented nearly half of all diabetic isolates (46.4%) but only 2.7% of non-diabetic isolates. Importantly, species-level identification of these isolates was confirmed using the API 20E and API 20 Strep systems, which ensured precise differentiation of closely related taxa (Table 2, Fig. 2).

**Table 2.** Bacterial isolates identified from gingivitis patients (by diabetic status)

Bacterial species	Non-diabetic (*n*=37 isolates)	%	Diabetic (*n*=28 isolates)	%
*Staphylococcus aureus*	15	40.5	8	28.6
*Streptococcus mutans*	17	45.9	6	21.4
*Bacillus* spp.	4	10.8	1	3.6
*Pseudomonas aeruginosa*	1	2.7	6	21.4
*Enterobacter aerogenes*	0	0.0	2	7.1
*Klebsiella pneumoniae*	0	0.0	1	3.6
*Escherichia coli*	0	0.0	2	7.1
*Proteus mirabilis*	0	0.0	2	7.1

n, number; %, percentage; spp., species.

Pearson’s *χ*² = 18.88, df=7, *P*=0.009. When aggregated, Gram-negative isolates (five taxa combined) were markedly enriched in diabetics (13/28, 46.4%) compared with non-diabetics (1/37, 2.7%): *χ*² = 15.54, df=1, *P*<0.001.

## Antimicrobial susceptibility

**Fig. 3.**
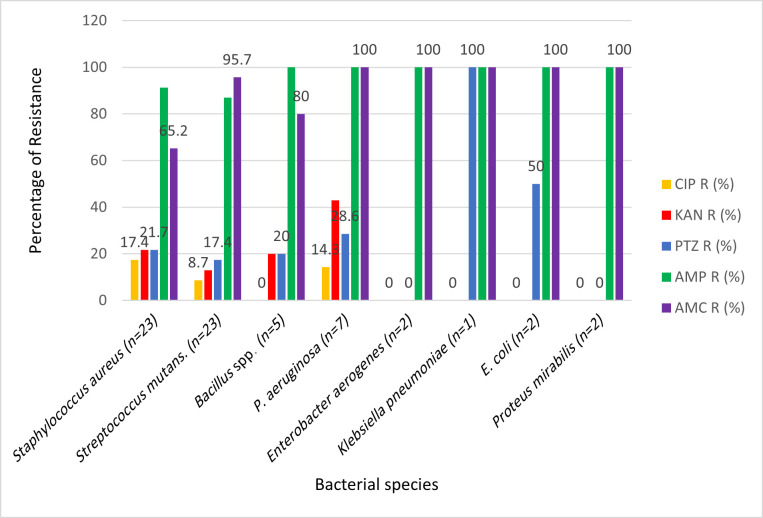
Antimicrobial susceptibility testing demonstrated higher resistance rates among isolates from diabetic patients across multiple antibiotic classes. In particular, resistance to *β*-lactams and aminoglycosides was markedly elevated (*P*<0.05, ANOVA). These findings highlight the increased antimicrobial resistance burden in the diabetic group.

Antibiotic resistance patterns also varied by species, as shown in Table 3 and Fig. 3. Non-diabetic-associated organisms (*Streptococcus mutans* and *Staphylococcus aureus*) showed high resistance to ampicillin (87–91 %) but generally retained good susceptibility to ciprofloxacin (82–91% susceptible) and kanamycin (78–87% susceptible).

**Table 3.** Antimicrobial resistance/susceptibility by species (counts with percentages)

Species	CIP R (%)	CIP S (%)	KAN R (%)	KAN S (%)	PTZ R (%)	PTZ S (%)	AMP R (%)	AMP S (%)	AMC R (%)	AMC S (%)
*Staphylococcus aureus* (*n*=23)	4 (17.4)	19 (82.6)	5 (21.7)	18 (78.3)	5 (21.7)	18 (78.3)	21 (91.3)	2 (8.7)	15 (65.2)	8 (34.8)
*Streptococcus mutans* (*n*=23)	2 (8.7)	21 (91.3)	3 (13.0)	20 (87.0)	4 (17.4)	19 (82.6)	20 (87.0)	3 (13.0)	22 (95.7)	1 (4.3)
*Bacillus* spp. (*n*=5)	0 (0.0)	5 (100)	1 (20.0)	4 (80.0)	1 (20.0)	4 (80.0)	5 (100)	0 (0.0)	4 (80.0)	1 (20.0)
*Pseudomonas aeruginosa* (*n*=7)	1 (14.3)	6 (85.7)	3 (42.9)	4 (57.1)	2 (28.6)	5 (71.4)	7 (100)	0 (0.0)	7 (100)	0 (0.0)
*Enterobacter aerogenes* (*n*=2)	0 (0.0)	2 (100)	0 (0.0)	2 (100)	0 (0.0)	2 (100)	2 (100)	0 (0.0)	2 (100)	0 (0.0)
*Klebsiella pneumoniae* (*n*=1)	0 (0.0)	1 (100)	0 (0.0)	1 (100)	1 (100)	0(0.0)	1 (100)	0 (0.0)	1 (100)	0 (0.0)
*Escherichia coli* (*n*=2)	0 (0.0)	2 (100)	0 (0.0)	2 (100)	1 (50.0)	1(50.0)	2 (100)	0 (0.0)	2 (100)	0 (0.0)
*Proteus mirabilis* (*n*=2)	0 (0.0)	2 (100)	0 (0.0)	2 (100)	0 (0.0)	2 (100)	2 (100)	0 (0.0)	2 (100)	0 (0.0)

AMC, amoxicillin–clavulanic acid; AMP, ampicillin; CIP, ciprofloxacin; KAN, kanamycin; PTZ, piperacillin–tazobactam; R, resistant; S, susceptible.

By contrast, Gram-negative bacilli recovered from diabetics displayed uniformly high resistance to *β*-lactams. All isolates of *Pseudomonas aeruginosa*, *Escherichia coli*, *Proteus mirabilis*, *Klebsiella pneumoniae* and *Enterobacter aerogenes* were resistant to both ampicillin and amoxicillin–clavulanic acid. Notably, *Klebsiella pneumoniae* (*n*=1) was resistant to every tested *β*-lactam and even to piperacillin–tazobactam, while *Escherichia coli* (*n*=2) exhibited resistance to *β*-lactams and partial resistance to piperacillin–tazobactam (50%).

Overall, across all species, ampicillin resistance exceeded 90%**,** and resistance to amoxicillin–clavulanic acid was above 80%. In contrast, non-*β*-lactam agents such as ciprofloxacin and kanamycin maintained comparatively better activity, with resistance rates of only 10–20%.

Overall comparison of resistance across antibiotics was statistically significant (*χ*² = 163.34, df = 4, *P* < 0.0001).

### *β*-Lactamase production

Enzymatic testing revealed *β*-lactamase activity in 44/65 isolates (67.7%)**,** with nearly identical prevalence between non-diabetic (67.6%) and diabetic (67.9%) groups.

**Fig. 4.**
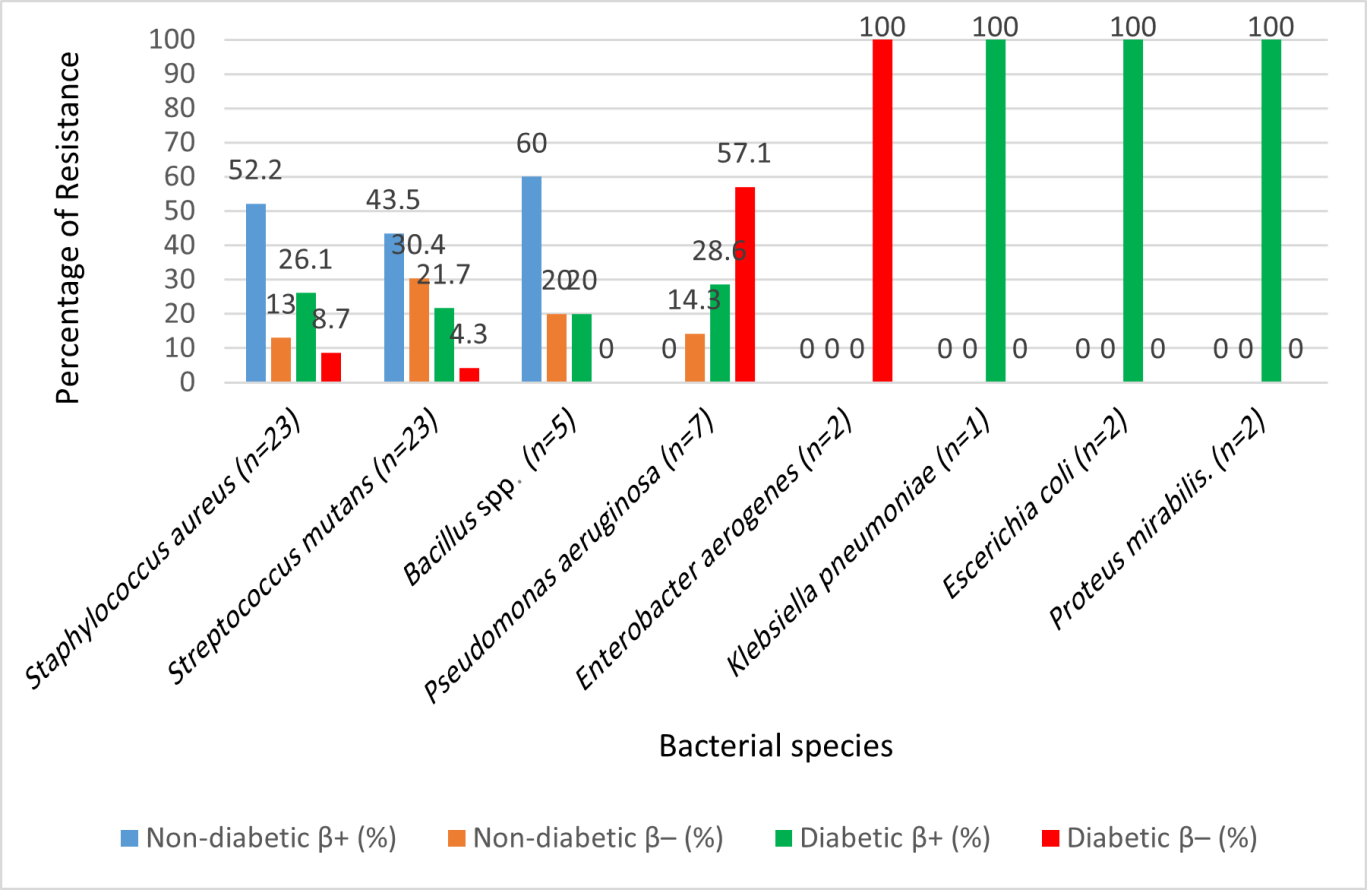
Quantitative analysis of *β*-lactamase activity revealed significantly elevated enzymatic activity in isolates from diabetic patients compared with non-diabetic patients (*P*<0.01, Student’s t-test). This further supports the role of diabetes in enhancing resistance mechanisms at the functional level.

At the species level, the highest positivity was seen in *Escherichia coli* (100%), *Proteus mirabilis* (100%) and *Klebsiella pneumoniae* (100%), all of which were exclusively recovered from diabetic patients. Among Gram-positive organisms, *Staphylococcus aureus* and *Streptococcus mutans* also showed high rates of *β*-lactamase production (52.2% and 43.5 %, respectively, in non-diabetics; 26.1% and 21.7% in diabetics). *Pseudomonas aeruginosa*, however, was negative for *β*-lactamase in all seven isolates as revealed in Table 4 and Fig. 4.

**Table 4.** β-lactamase production by species and diabetic status (counts with percentages)

Species	Non-diabetic β+ (%)	Non-diabetic β– (%)	Diabetic β+ (%)	Diabetic β– (%)
*Staphylococcus aureus* (*n*=23)	12 (52.2)	3 (13.0)	6 (26.1)	2 (8.7)
*Streptococcus mutans* (*n*=23)	10 (43.5)	7 (30.4)	5 (21.7)	1 (4.3)
*Bacillus* spp. (*n*=5)	3 (60.0)	1 (20.0)	1 (20.0)	0 (0.0)
*Pseudomonas aeruginosa* (*n*=7)	0 (0.0)	1 (14.3)	2 (28.6)	4 (57.1)
*Enterobacter aerogenes* (*n*=2)	0 (0.0)	0 (0.0)	0 (0.0)	2 (100)
*Klebsiella pneumoniae* (*n*=1)	0 (0.0)	0 (0.0)	1 (100)	0 (0.0)
*Escherichia coli* (*n*=2)	0 (0.0)	0 (0.0)	2 (100)	0 (0.0)
*Proteus mirabilis* (*n*=2)	0 (0.0)	0 (0.0)	2 (100)	0 (0.0)

n, number; %, percentage; *β*+, *β*-lactamase-positive; *β*−, *β*-lactamase-negative.

Fisher’s exact test yielded *P*=1.000, indicating no significant difference in overall *β*-lactamase positivity between diabetic and non-diabetic isolates. Despite the Gram-negative shift and higher polymicrobial infections in diabetics, overall *β*-lactamase positivity did not differ significantly between groups.

## Discussion

This study investigated the prevalence and characteristics of *β*-lactamase-producing bacteria in gingivitis patients with and without diabetes. The findings demonstrate clear differences in the microbial composition and resistance patterns between the two groups, underscoring the potential impact of diabetes on the oral microbiome and antimicrobial resistance.

### Influence of age on microbial distribution

Age exerts a profound influence on the composition and pathogenic potential of the oral microbiota. Younger individuals often exhibit higher levels of cariogenic species such as *Streptococcus mutans*, reflecting dietary sugar consumption, immature oral hygiene behaviours and greater enamel susceptibility [15]. In contrast, older individuals demonstrate ecological shifts characterized by gingival recession, reduced salivary flow and immune senescence, all of which create niches conducive to colonization by opportunistic Gram-negative organisms such as *Pseudomonas aeruginosa* and *Enterobacteriaceae* [16,17]. In diabetic patients, this age-related vulnerability is compounded by hyperglycaemia and impaired neutrophil function, resulting in increased microbial diversity and elevated risk of colonization by *β*-lactamase-producing bacteria [18]. These findings highlight the need to interpret microbial differences not only in the context of diabetic status but also age-related ecological changes.

### Polymicrobial infections

Polymicrobial infections occurred more frequently among diabetic patients than non-diabetic counterparts, consistent with the concept that hyperglycaemia favours microbial co-aggregation and biofilm complexity [19]. Previous studies demonstrated that high glucose concentrations in gingival crevicular fluid promote the growth of both facultative and obligate anaerobes, leading to synergistic microbial interactions that exacerbate gingival inflammation [20,21]. Moreover, immunological dysregulation in diabetes reduces the host’s ability to contain mixed-species biofilms, explaining the greater polymicrobial burden observed in this group [22]. This observation aligns with reports from periodontal studies in Saudi Arabia and India, where diabetic patients consistently demonstrated higher rates of polymicrobial colonization than non-diabetic controls [23,24]. Clinically, such polymicrobial profiles complicate treatment, as interspecies communication within biofilms increases tolerance to antimicrobials and fosters horizontal gene transfer of resistance determinants [25].

### Distribution of bacterial species

The bacterial spectrum observed in this study reflects both typical oral commensals and opportunistic pathogens. *Streptococcus mutans* and *Staphylococcus aureus* predominated in non-diabetic patients, consistent with earlier findings that these species dominate in caries-prone and gingivitis-associated sites [26,27]. In contrast, diabetic patients showed a higher prevalence of *Pseudomonas aeruginosa*, *Enterobacter aerogenes* and *Escherichia coli*, organisms rarely found in healthy oral cavities but increasingly recognized as opportunistic colonizers in systemic disease [28].

The predominance of *Staphylococcus aureus* is notable given its dual role as a commensal and opportunistic pathogen. Its ability to produce *β*-lactamase, survive within host cells and form robust biofilms provides competitive advantages in the diabetic oral niche [29]. Similarly, the detection of *Bacillus* spp. underscores the diverse microbial ecosystem in gingivitis, though their pathogenic contribution remains debated [30].

The isolation of *Enterobacteriaceae* such as *Escherichia coli *and *Klebsiella pneumoniae* in diabetic patients mirrors findings from Egyptian and Turkish cohorts where such species were linked with poor glycaemic control and advanced periodontal disease [31,32].

The ecological shift toward Gram-negative opportunists in diabetics reflects metabolic dysfunction, reduced salivary flow and impaired mucosal immunity, all of which lower the threshold for colonization by non-oral bacteria [33].

### Antimicrobial resistance patterns

The antimicrobial resistance patterns observed highlight an alarming decline in susceptibility among oral isolates. High resistance to ampicillin and amoxicillin-clavulanate among *Staphylococcus aureus* and *Pseudomonas aeruginosa* mirrors regional reports from North Africa and the Middle East [34,35]. This pattern likely reflects indiscriminate antibiotic use, often without prescription, in community settings [36]. The relatively preserved susceptibility to ciprofloxacin and piperacillin–tazobactam suggests that these agents remain partially effective; however, reliance on them risks accelerating resistance development [37]. Of particular concern is the detection of multidrug-resistant *Pseudomonas aeruginosa*, which has been linked to biofilm-associated infections and therapeutic failures in diabetic populations [38]. Comparisons with global surveillance studies confirm that resistance rates among oral isolates are rising, raising questions about the oral cavity as a reservoir of resistant genes [39,40]. This resistance profile emphasizes the need for local antimicrobial stewardship, routine surveillance of oral isolates and updated clinical guidelines for treating diabetic patients with periodontal disease [41].

### *β*-Lactamase production

The identification of *β*-lactamase-producing strains among both Gram-positive and Gram-negative bacteria is of particular clinical concern. In diabetics, the higher prevalence of *β*-lactamase production aligns with evidence that hyperglycaemia promotes bacterial stress responses and plasmid stability, both of which enhance resistance gene persistence [42]. Reports from Libyan, Indian and European cohorts confirm that *β*-lactamase activity is increasingly common in oral pathogens, particularly in *Staphylococcus aureus*, *Klebsiella pneumoniae* and *Escherichia coli* [43,45]. Although molecular assays such as PCR would provide more definitive characterization, the phenotypic detection used here offers valuable preliminary insights. Importantly, *β*-lactamase production contributes to therapeutic failures in odontogenic infections, necessitating cautious antibiotic selection [46]. The findings, therefore, emphasize that empirical use of *β*-lactams in gingivitis patients, particularly those with diabetes, may be inadequate and require consideration of *β*-lactamase-stable or combination regimens [47].

### Clinical implications

The interplay between diabetes, oral microbiota and antimicrobial resistance has significant clinical consequences. The increased polymicrobial burden, higher prevalence of opportunistic Gram-negative bacteria and elevated *β*-lactamase production together suggest that diabetic patients are at greater risk of treatment failure and progression from gingivitis to periodontitis [48]. These findings support recommendations for targeted oral health interventions in diabetics, including rigorous plaque control, regular dental monitoring and judicious antibiotic use guided by culture and sensitivity testing [49,50]. Moreover, the identification of resistant Gram-negative organisms underscores the oral cavity’s role as a potential reservoir for nosocomial pathogens, extending the implications beyond dentistry to systemic health [51].

## Conclusion

Within the limits of this small-scale study, diabetic patients with gingivitis appeared to harbour a more diverse microbial profile with frequent *β*-lactamase-producing bacteria compared with non-diabetics. The isolation of *Streptococcus mutans* and *Staphylococcus aureus* aligns with their known role in gingival disease, while opportunistic Gram-negative bacilli in diabetics likely reflect altered immunity and hyperglycaemia. The observed resistance to ampicillin and amoxicillin–clavulanate highlights a therapeutic challenge and supports integrating microbial and resistance profiling into periodontal management, especially in diabetic patients.

### Limitations

This study was limited by its relatively small sample size and resource-intensive microbiological methods, which restrict the generalizability of the findings. In addition, no patient outcome data were collected, preventing assessment of the microbiological findings in relation to treatment response. Finally, molecular diagnostic techniques were not employed due to resource constraints; reliance on phenotypic methods may have underestimated microbial diversity and resistance mechanisms [52].

### Recommendations and future directions

Further research with larger, multicentre populations is warranted to confirm these findings. Incorporating molecular techniques would enable more accurate detection of microbial diversity and resistance genes. Clinically, diabetic patients may benefit from closer microbiological monitoring in cases of recurrent gingival infection. Strengthening antibiotic stewardship and conducting longitudinal studies linking glycaemic control, microbial composition and periodontal outcomes could provide valuable insights for prevention and treatment strategies [53,54].

## Abbreviations

API: analytical profile index
